# Icing Condition Predictions Using FBGS

**DOI:** 10.3390/s21186053

**Published:** 2021-09-09

**Authors:** Miguel González del Val, Julio Mora Nogués, Paloma García Gallego, Malte Frövel

**Affiliations:** 1 Instituto Nacional de Técnica Aeroespacial (INTA), Crta. de Ajalvir km 4, 28850 Torrejón de Ardoz, Spain; garciagp@inta.es (P.G.G.); frovelm@inta.es (M.F.); 2 Ingeniería de Sistemas para la Defensa de España S.A. (ISDEFE), C. de Beatriz de Bobadilla, 3, 28040 Madrid, Spain; modsurf4.pers_externo@inta.es; 3 Escuela Internacional de Doctorado, Universidad Nacional de Educación a Distancia (UNED), Calle de Bravo Murillo, 38, 28015 Madrid, Spain

**Keywords:** ice detection, FBGS, icing, aircraft, fiber optic, ice modeling, latent heat, temperature, SLD

## Abstract

Icing is a hazard which is important for the aerospace industry and which has grown over the last few years. Developing sensors that can detect the existence not only of standard icing conditions with typically small droplet size, but also of Supercooled Large Droplet (SLD) conditions is one of the most important aims in order to minimize icing hazards in the near future. In the present paper a study of the Fiber Bragg Grating Sensors’ (FBGSs) performance as a flight icing detection system that predicts the conditions of an icing cloud is carried out. The test matrix was performed in the INTA Icing Wind Tunnel (IWT) with several icing conditions including SLD. Two optic fibers with 16 FBGS in total were integrated in the lower and upper surface of an airfoil to measure the temperature all over the chord. The results are compared with a Messinger heat and mass balance model and the measurements of the FBGS are used to predict the Liquid Water Content (LWC) and Ice Accretion Rate (IAR). Finally, the results are evaluated and a sensor assessment is made. A good correlation was observed between theoretical calculations and test results obtained with the FBGS in the IWT tests. FBGS proved to detect the beginning and end of ice accretion, LWC and IAR quickly and with good precision.

## 1. Introduction

Atmospheric icing occurs when aircraft fly through a cloud with supercooled droplets. Currently, there is a need for sensors that can detect and evaluate the hazard in a short time and in a feasible way. Actual sensors used in aircrafts (A/C) such as the magnetostrictive sort [1] have proven to work reliably in standard icing conditions described in the aviation safety standards FAR 25 Appendix C (App.C) [2] where small droplets with a Median Volume Diameter (MVD) of 10 to 40 microns are present. Many sensors can only alert users to the fact that icing in this condition has started but cannot evaluate the icing condition with its MVD and Liquid Water Content (LWC) [1]. Up to now that was sufficient to satisfy the A/C safety. Commercial aircraft is certified against App. C condition and can fly in this condition for a predefined time using its counter measures against icing. Nowadays, the A/C safety requirements ask for sensors that are also able to detect big and Super Large Droplets (SLD) in access of 100 microns MVD which has shown to be very dangerous for A/C safety, although they normally occur only relatively seldomly. These dangerous icing conditions, addressed in Appendix O (App. O) of the FAR25 [3], require new sensors that are able to differentiate when the supportable App. C condition is present or when the hazardous App. O condition starts and special measures need to be taken. The App. O requirements make very important the development of sensors that can evaluate SLD conditions and give as much information about MVD, LWC and ice accretion rate (ACR) as possible.

Several Flight Icing Detection Systems (FIDSs) have been developed (Jackson [1]) but in this paper a latent heat based sort is going to be studied that uses optic fiber Bragg grating sensors (FBGSs) and measures temperature changes produced by the instant phase change of liquid supercooled droplets to ice. The first heat transfer based sensor was patented by Tribus and Moyle [4] in 1956. The apparatus was based on detecting the voltage difference between two thermocouples, one of them in contact with the air flow. FBGSs have the advantage over thermocouples that they are immune against electromagnetic interference; they have very low mass, fast response and very low heat conduction, so they can give a lot of information about the thermodynamic events during a test. An FIDS using FBGS has been developed by Frövel et al. [5]. In this patent an FBGS is embedded in an airfoil. In the optic fiber several FBGSs are located along the chord for knowing the temperature in all the airfoil.

Many heat transfer studies have been carried out in order to predict the ice accretion in airfoils. Messinger [6] did a heat balance study introducing the concept of freezing fraction which was developed by Ruff [7]. Those concepts were used in much ice prediction software like LEWICE [8,9]. In the case of large droplets, new models have been made [10]. Myers [11] made a model that takes conduction into account.

An important issue of modelling heat transfer is the convective heat transfer coefficient calculation. On turbulent flow, the heat transfer coefficient depends on the equivalent sand-grain roughness height [8,12]. The equivalent sand-grain roughness height calculation has been considered a too complex approximation for this problem [13].

In this work a model for the prediction of the icing cloud conditions, heat transfer coefficient and ice accretion rate is presented based on the models from the LEWICE software [8] and G. Fortin [13]. The theoretical results are compared with experimental data obtained with the FIDS from Frövel et al. The tests were performed in the INTA icing wind tunnel (IWT) in typical aeronautical icing conditions.

## 2. Materials and Methods

### 2.1. Fiber Bragg Grating as a Temperature Sensor

Firstly, it is important to describe briefly all the components involved in the tests. One of them is the Fiber Bragg Grating Sensors (FBGSs) from the company FBGS INTERNATIONAL, manufactured in the so-called draw tower process coated with ORMOCER [14] and with a typical fiber diameter of 125 microns. These so-called Draw Tower Gratings (DTGs) are high resistant sensors that present almost the same breaking strength values as the optical fiber without any grating. The FBGS optic fiber sensors use the principle of Bragg reflection in a specific broadband.The reflected wavelength is a function of variables such as temperature or stress [15]. Reference [16] shows an equation that relates temperatures and wavelength ($\lambda_{B}$). Equation (1) shows a relationship between wavelength, temperature, strain (ε) and refractive index (*n*) [17]:

(1) $$\lambda_{B}\left(\varepsilon ,T\right)=2n\left(T\right)\Lambda \left(T,\varepsilon \right)$$

In order to isolate the FBGS from strains (ε), the optic fiber was introduced in a capillary, so Equation (1) can be simplified as a third degree polynomial function $T\left(\lambda_{B}\right)=a\lambda_{B}^{3}+b\lambda_{B}^{2}+c\lambda_{B}+d$ [17,18] (Reference [17] Section 8.2 provides a table with different thermal expansion and thermo-optic coefficients depending on the temperature; it can be seen that the thermal expansion coefficient is almost constant at temperatures higher than 250 K and the thermo-optic can be expressed as a polynomial). The curve is obtained by a calibration process using a SIKA TP3M165E2 highly precise temperature calibrator with an accuracy of 0.3 ${}^{\circ }$C and stability of 0.01 ${}^{\circ }$C. For this calibration, the instrumented airfoil was introduced in the temperature calibrator and three cycles with five temperature steps were applied between 20 ${}^{\circ }$C and −30 ${}^{\circ }$C. In order to determine the accuracy of the calibration, the average value of the standard deviation of the grating values is obtained (Equation (3). The accuracy of the sensors at the temperature *T* is $acc_{T}$. The number of gratings is $N_{gratings}$ and the standard deviation of the wavelength values in the three cycles at temperature *T* is $\lambda_{grating}^{T}$. The accuracy results are represented in Table 1:

(2) $$std\left(x\right)=\sqrt{\frac{\sum_{cycle=1}^{3}\left(x_{cycle}-\bar{x}\right)}{3}}$$

(3) $$acc_{T}=\frac{1}{N_{gratings}}\sum_{grating=1}^{N_{gratings}}std\left(\lambda_{grating}^{T}\right)$$

As previously mentioned, for isolation reasons and to avoid any parasitic strain, the optic fiber is located in a freely movable manner inside a polyimide capillary with an external diameter of 0.55 mm. The capillary is filled with silicone oil to enhance the thermal heat transfer from the sensor surface to the sensor and, this way, its response time is improved. The sensor zones were chemically stripped in a sulfuric acid bath to remove the coating and guarantee a high precision of the sensors’ temperature measurements by avoiding any influence of a coating material. The distance between gratings was one centimeter and the width of the grating Λ was 8 mm. A commercial optical interrogator was used, the Luna Hyperion si155, with a wavelength range of 1500 to 1600 nm and an accuracy of 1 pm. The si155 features a high power, wide swept wavelength laser with guaranteed absolute accuracy on every scan, which is realized with Micron Optics patented Fiber Fabry-Perot filter and wavelength reference technology. The interrogator counts on rapid, full-spectrum data acquisition and flexible peak detection algorithms of FBGSs.

**Figure 1:**
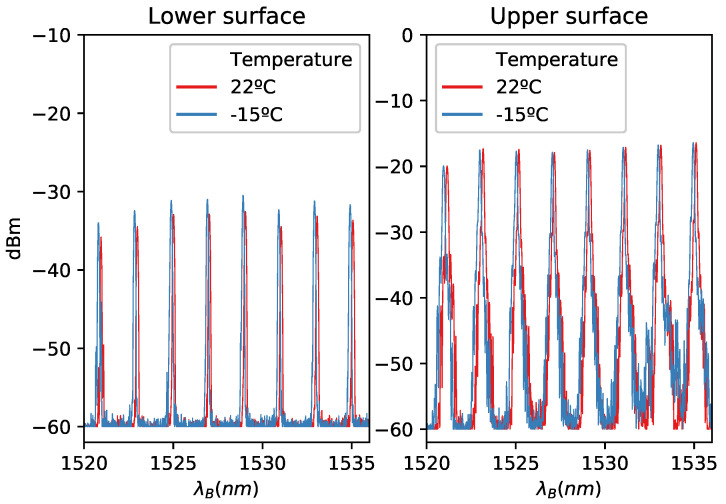
Spectral response of the sensor for two different temperatures.

The spectral response of the fiber can be seen in Figure 1. The spectral response of the lower surface is weaker than the spectral response of the upper surface, but all peaks can be detected without problems. Both responses were represented for different temperatures, 22 ${}^{\circ }$C and −15 ${}^{\circ }$C, before and during icing, respectively.

### 2.2. Model Resolution

**Figure 2:**
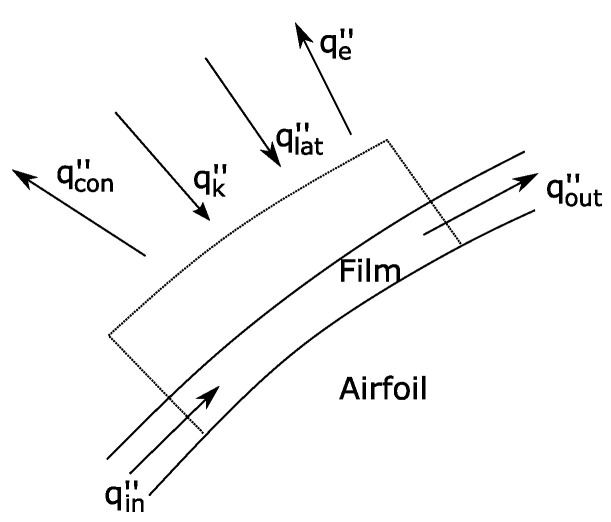
Heat Transfer Control Volume.

**Figure 3:**
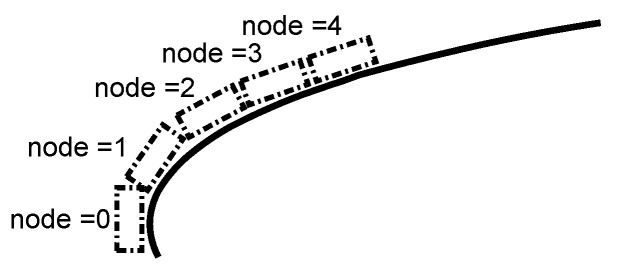
Airfoil surface discretization.

In order to predict the ice conditions, it is important to define a model. For modelling the temperature in the surface of the airfoil, the heat (5) and mass balance (4) equations of reference [8] are used. The control volume used is represented in Figure 2. It represents the initial control volume previous to the ice accretion. The airfoil surface is divided into nodes, and each one represents a control volume node, as can be seen in Figure 3. Normally, the modelled temperature in the ice accretion codes is the ice surface temperature, but in this document it is considered that initially the ice surface temperature is similar to the airfoil surface temperature. The airfoil has been made in Polylactic Acid (PLA) using additive manufacturing. The PLA thermal conductivity is less than ice, so the conduction heat flux is negligible (normally in the icing accretion codes [8], conduction is considered negligible when an ice layer is formed; PLA has lower thermal conductivity than ice so this hypothesis could be applied in the present case).

(4) $${\dot{m}}_{ice}^{node}={\dot{m}}_{im}^{node}+{\dot{m}}_{rb}^{node}-{\dot{m}}_{out}^{node}-{\dot{m}}_{evap}^{node}$$

In Equation (4), ${\dot{m}}_{ice}^{node}$ is the mass flux of ice formed in the surface, ${\dot{m}}_{im}^{node}$ is the mass flux of water that impinges the surface, ${\dot{m}}_{rb}^{node}$ is the mass flux that enters in the control volume represented in Figure 2, ${\dot{m}}_{out}^{node}$ is the mass flux that leaves that control volume and ${\dot{m}}_{evap}^{node}$ is the mass flux of water that is evaporated.

(5) $$F\left(f^{node},T_{sur}^{node}\right)=q_{lat}^{node}-q_{evap}^{node}\pm q_{sens}^{node}+q_{k}^{node}-q_{nc}^{node}=0$$

The first term in the energy balance equation represents the fusion of impinging water which is the product between the impinging water mass flux (${\dot{m}}_{im}$) and the water latent energy of fusion ($L_{f}$). The impinging water mass flux is proportional to the collection efficiency value β, the Liquid Water Content (LWC) and the airspeed ($V_{\infty }$):

(6) $$q_{lat}^{node}={\dot{m}}_{im}^{node}L_{f}=\beta^{node}V_{\infty }LWCL_{f}$$

The second term represents the evaporation of a small liquid mass in the air. It is produced due to the water vapour concentration difference between the surface and the air. The evaporative flux heat is proportional to the evaporative mass flux and to the latent heat of vaporization ($L_{v}$). The mass flow rate between the surface and the air (${\dot{m}}_{evap}$) could be expressed as [13]:

(7) $$q_{evap}={\dot{m}}_{evap}^{node}L_{v}=h_{g}^{node}\left(\rho_{vs}^{node}-\rho_{ve}^{node}\right)L_{v}$$

The evaporative term is only taken into account in case of glaze ice, because in rime ice there is not a water film that flows on the airfoil surface. The water vapour densities at the surface $\rho_{vs}$ and in the air $\rho_{ve}$ are expressed as functions of the saturated vapour pressure $P_{vs}$ at the surface and in the airflow and the relative humidity ϕ (G. Fortin 2006 [13]).

(8) $$\rho_{vs}^{node}=\frac{P_{vs}\left(T_{sur}^{node}\right)}{R_{v}T_{sur}^{node}}$$

(9) $$\rho_{ve}^{node}=\phi \frac{P_{vs}\left(T_{e}^{node}\right)}{R_{v}T_{e}^{node}}$$

(10) $$P_{vs}\left(T\right)=610.8e^{\frac{L_{v}}{R_{v}}\left(T_{f}^{-1}-T^{-1}\right)}$$

The convective mass transfer coefficient $h_{g}$ [8] is expressed as a function of the Lewis number (Le) and the convective transfer coefficient ($h_{con}$). The Lewis number is the ratio between the thermal and mass diffusivity $D_{av}$:

(11) $$h_{g}^{node}=\frac{h_{con}^{node}}{\rho_{a}c_{p,a}Le^{2/3}}$$

(12) $$Le^{node}=\frac{k_{a}^{node}}{\rho_{a}c_{p,a}D_{av}\left(T_{film},p_{st}\right)}$$

The third term represents the sensible heat of water and ice. In this term, the sensible heat of water and of ice on the surface of the impinging ($q_{sens,im}$) and the incoming water ($q_{rb,im}$) mass flow are included:

(13) $$q_{sens}^{node}=q_{sens,im}+q_{sens,rb}$$

The sensible heat of impinging water depends on the impinging water mass flow and of the specific heat of water in the airfoil surface ($c_{p,w}$) and of ice ($c_{p,i}$):

(14) $$q_{sens,in}=-{\dot{m}}_{im}^{node}\left(cp_{w}^{node}\left(T_{mp}-T_{st}^{node}\right)+c_{p,i}^{node}\left(T_{sur}^{node}-T_{mp}\right)\right)$$

Additionally, the sensible heat of incoming water depends on the specific heat of water in the previous control volume $c_{p,w}^{node-1}$ and of the specific heat of ice.

(15) $$q_{sens,rb}=-{\dot{m}}_{rb}^{node}\left(c_{p,w}^{node-1}\left(T_{mp}-T_{sur}^{node-1}\right)+c_{p,i}\left(T_{sur}^{node}-T_{mp}\right)\right)$$

The fourth term represents the heat flow gained by the surface due to the kinematic energy of the incoming droplets:

(16) $$q_{k}^{node}={\dot{m}}_{im}\frac{V_{\infty }^{2}}{2}$$

The last term represents the convective heat transfer. Normally, it is defined as the net convective loss from the body (Equation (19)) which is the difference between the convective heat lost and the frictional heat gained [9]. The convective heat flux transfer is proportional to the difference between the airfoil surface temperature $T_{sur}$ in the control volume and the static temperature $T_{\infty }$. It is highly dependant on the convective heat transfer coefficient.

(17) $$q_{con}^{node}=h_{con}^{node}\left(T_{sur}^{node}-T_{\infty }\right)$$

The frictional gained heat flux is proportional to the difference between the recovery temperature $T_{rec}$ and the static air temperature $T_{\infty }$

(18) $$q_{fric}^{node}=h_{con}^{node}\left(T_{rec}^{node}-T_{\infty }\right)$$

(19) $$q_{nc}^{node}=q_{con}^{node}-q_{fric}^{node}=h_{con}^{node}\left(T_{sur}^{node}-T_{rec}^{node}\right)$$

For solving the energy balance equation, the collection efficiency and convective heat transfer coefficient must be calculated. Both parameters depend on the fluid field which is calculated with a CFD model (Section 2.2.1). Once the fluid field has been solved, the laminar convective heat transfer coefficient (Section 2.2.2) and the collection efficiency (Section 2.2.3) are calculated as well. Finally, an energy balance resolution algorithm is used for obtaining the temperature profile in the airfoil (Section 2.2.4).

#### 2.2.1. CFD Model

**Figure 4:**
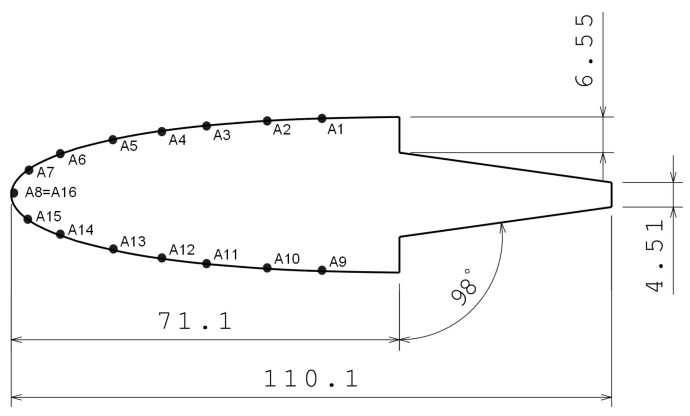
Airfoil dimensions.

The air stream around the airfoil was modelled using OpenFoam. Normally, in ice accretion codes, a potential flow model is used for solving the airflow. Due to the anomalous airfoil used for the tests (Figure 4) a turbulent Spalart–Allmaras model [19] was considered. The selected kinematic viscosity is 12.43 × 10${}^{-6}$ m${}^{2}$/s. The rest of the constant values are similar to reference [20]. All the conditions of the model are exposed in Table 2.

#### 2.2.2. Convective Heat Transfer Coefficient

One of the most important parameters in predicting ice accretion behaviour is the convection. Many efforts have been made to determine the values of the convective heat transfer coefficient Samad, A [21]. For calculating the heat transfer coefficient, a laminar integral boundary layer method has been used [22], with $\delta_{T}$ being the thickness of the thermal boundary layer and $k_{a}$ the thermal conductivity of air (Equation (20)). The result can be expressed as a function of the external flow temperature $V_{e}$ [9]:

(20) $$h_{l}=\frac{2k_{a}}{\delta_{T}}$$

The thermal boundary layer thickness can be expressed as a function of the surface distance from the stagnation point (*s*):

(21) $$\delta_{T}\left(s\right)=\frac{46.72\nu_{a}}{V_{e}{\left(s\right)}^{2.87}}\int_{0}^{s}V_{e}{\left(x\right)}^{1.87}dx$$

The integral will be solved using the trapezoidal rule. The external airspeed was selected taking a vector with a direction that is normal concerning the airfoil surface and its modulus is a distance that satisfies the condition that the exterior airspeed is constant in the surface normal direction.

Another way to calculate the convective heat transfer coefficient in the stagnation point is by using Equation (22). According to the reference [23], the heat transfer along a cylinder in the stagnation line is a function of the air density $\rho_{a}$, air viscosity $\mu_{a}$, airspeed $U_{\infty }$, Prandtl number Pr, leading edge equivalent diameter *D* and thermal conductivity of air:

(22) $$h_{c}=1.14{\left(\frac{\rho_{a}V_{\infty }D}{\mu_{a}}\right)}^{0.5}Pr^{0.4}\frac{k_{a}}{D}$$

For an airfoil an equivalent diameter of the leading edge is calculated. The NACA 0012 leading edge equivalent diameter *D* is a 3.16% of the chord [24] of the original airfoil.

#### 2.2.3. Collection Efficiency Calculation

The value of the impinging water mass depends on the local collection efficiency β(x). Using a Lagrangian perspective, the local collection efficiency is calculated integrating trajectories of a droplet population distribution (Chang et al. [25]). The local collection efficiency was calculated with Equation (23) and the droplets’ trajectories were integrated according to the equations of reference Zarling [26] and using a Euler method. In Equation (23), the term $dy_{0}$ represents the variation between the droplet initial and final heights and ds represents the airfoil distance between the airfoil point where the *y* component is equal to the initial point of the droplet trajectory and the last point of the integrated trajectory [8].

(23) $$\beta {\left(s\right)}^{dropsize}=\frac{dy_{0}}{ds}$$

Once the collection efficiency of each droplet size has been calculated, their weighted average with the volume percentage is made. In this paper it has been considered that all the droplet population collection efficiencies can be approximated as a unique droplet trajectory where the diameter value is equal to the MVD of the droplet population (without this simplification the problem would be different for each droplet distribution).

#### 2.2.4. Model Resolution

**Figure 5:**
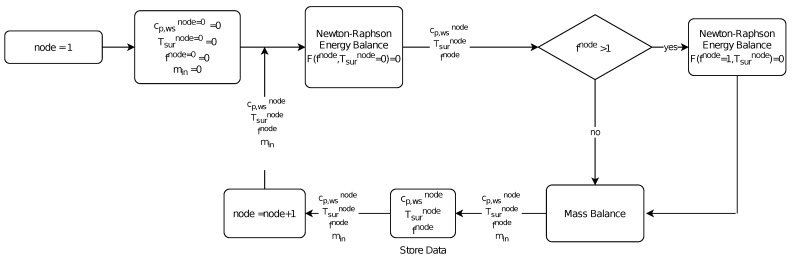
Algorithm resolution scheme.

The model algorithm resolution begins in the stagnation point node (node 1 in Figure 5). In the stagnation node, the mass flow of incoming water does not exist. The resolution is made in two steps [8]. Firstly, glaze ice conditions are assumed. In glaze ice, the freezing fraction is less than one and the surface temperature is the same as the melting point temperature [6]. If the mathematical solution of the freezing fraction with a glaze hypothesis is higher than the unity, the previous assumption is wrong, so the model would be resolved with a rime supposition. In the case of being less than one, the hypothesis was right, so the water flow mass that is going out of the control volume is calculated using the mass balance equation. In glaze ice, the equation is non-linear, so the Newton–Raphson method is used in order to calculate the freezing fraction of each node.

In the case of rime ice, the surface temperature is less than the melting temperature and the freezing fraction is one. In rime ice, the evaporative term is null ( Messinger [6]), so the energy balance equation is linear. Once a feasible result has been obtained, the same process is done with the following node (see Figure 5).

### 2.3. Tests Description

Several tests at different temperatures and ordered in a test matrix were carried out in order to obtain repetitive results. The tests were planned to have outcomes in two types of ice—glaze and rime. All tests were recorded with the same time sequence for having a visual explanation of the sensor signals and for giving sense to the signal events recorded.

The test matrix was designed with the goal of comparing the results with a constant parameter in different conditions of LWC and MVD. Each test matrix has constant ambient temperature and a fixed air speed of 70 m/s. Two matrices were tested, one at −5 ${}^{\circ }$C and the other at −13.5 ${}^{\circ }$C. Conditions are described in Table 3. There is one Appendix O [3], freezing drizzle conditions, with LWC ≈ 0.3 g/m${}^{3}$ and with MVD of 40 μm.

The airfoil used in the tests is a NACA 0012, cut for not having a too large chord for testing in the icing tunnel. The dimensions of the airfoil are shown in Figure 4, with sensors uniformly separated all over the chord of the airfoil. This airfoil was chosen due to its thickness and because of its symmetry. Several tests with different airfoils were done previously in order to find out the effect of the airfoil thickness in ice formation with SLD conditions. A NACA 0012 was selected for these tests because it was observed that in airfoils with less thickness the large droplets impinge farther from the leading edge.

In order to detect ice formation and to obtain information about the temperatures in different locations all over the chord, eight sensors were placed in the upper surface and another eight in the lower surface. Of those, four were placed in the leading edge, two in the upper and another two in the lower surface. Leading edge sensors have a big temperature step in the conditions of Table 3 so they are very useful for ice detection. The other sensors can be useful in order to know the environmental icing conditions. Positions and names of each sensor are in Table 4.

**Figure 6:**
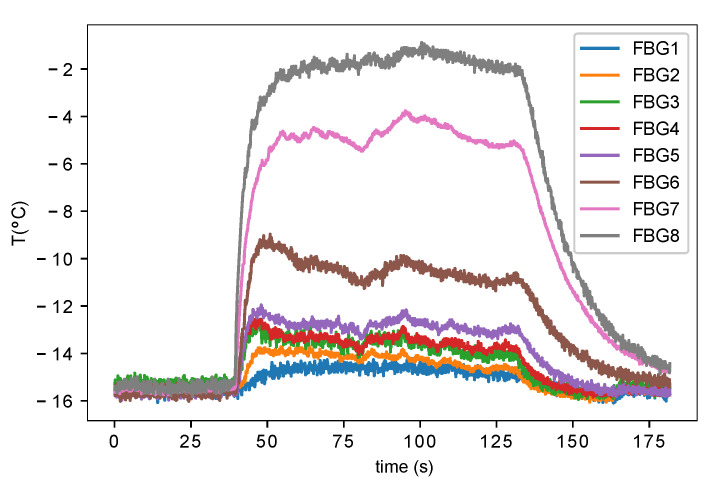
Test structure example. Angle of attack: 10${}^{\circ }$. $V_{TS}$ = 70 m/s.

Every test consists of one icing cycle of approximately 90 s. An example of the signals of the tests can be seen in Figure 6. The tests have three stages:

1. The temperature is stabilised in an equilibrium temperature before the fogging.
2. In the 40th second the temperature rises abruptly due to the beginning of the icing cycle. It stabilizes in a new equilibrium temperature.
3. In the 130th second the fogging stops and the temperatures drop abruptly.

## 3. Results

### 3.1. Ice Model Validation

**Figure 7:**
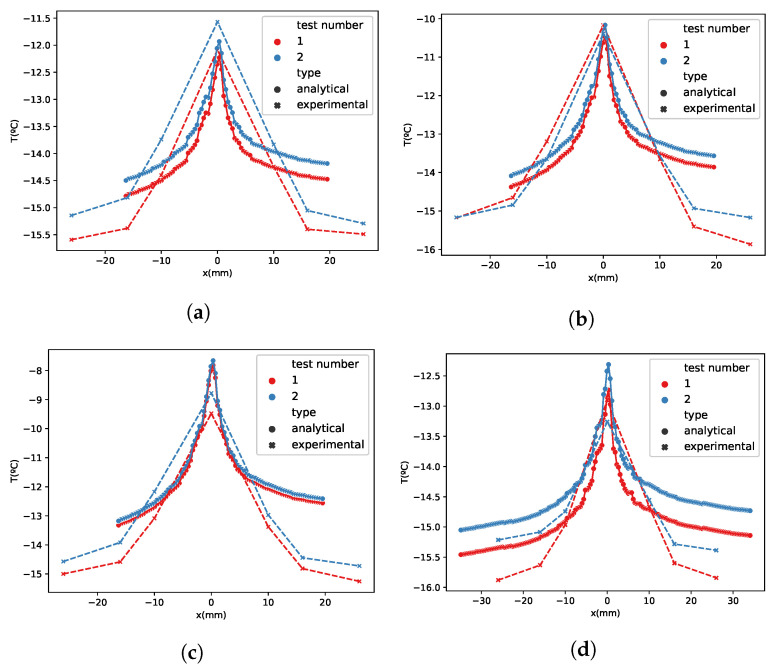

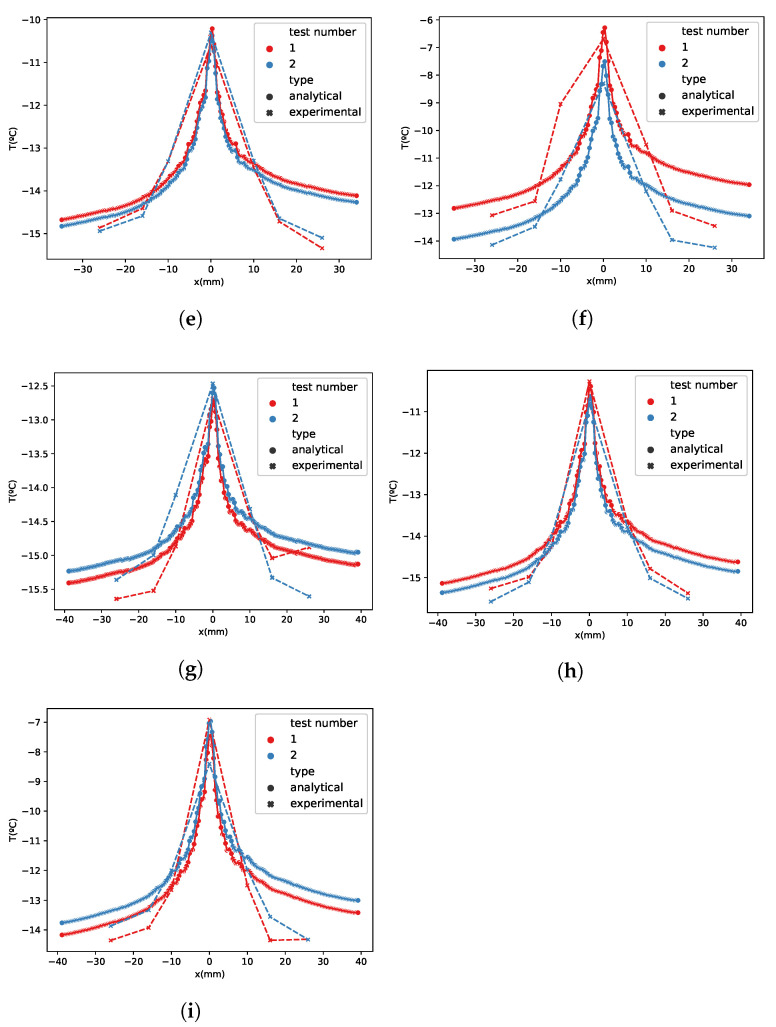
Rime test matrix. Angle of attack: 0${}^{\circ }$. Test cases: (**a**) 10 (**b**) 13 (**c**) 16 (**d**) 11 (**e**) 14 (**f**) 17 (**g**) 12 (**h**) 15 (**i**) 18.

Several tests were carried out in order to validate the thermal model (Equation (5)). The first model will be in rime, zero degrees of angle of attack, seventy meters per second of airspeed and with the cloud conditions described in Table 3. The results of the model can be seen in Figure 7. Two repetitions in rime ice with 0${}^{\circ }$ angle of attack were conducted: The first repetition in blue and the second one in red. The analytical model results in continuous lines and the experimental one results in discontinuous lines. The model seems accurate close to the leading edge but there is an increase in the error downstream from the leading edge. This is because of the turbulence generated by the ice roughness. There are many models that try to simulate the roughness of the ice depending on many parameters (G. Fortin [13]), but they are difficult to implement, so in this case they have not been used to keep the problem simple.

**Figure 8:**
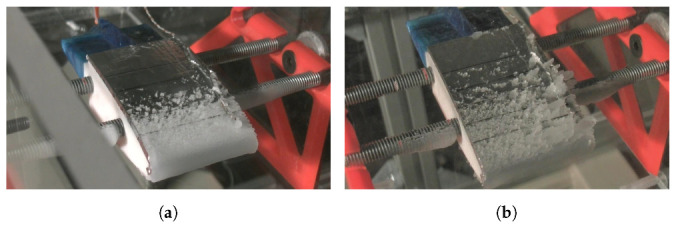
Pictures taken from IWT tests with: **a** MVD=20μm (Test case 13). (**b**) MVD=70μm (Test case 15).

Another aspect to take into account is the similarity between Figure 7 curves and a typical local collection efficiency curve. In case of rime ice, the impinging water function is proportional to the local collection efficiency, and the latent heat term of the energy balance equation is the most relevant, so the collection efficiency is an important parameter in order to determine the temperature profiles. In the analytical results of Figure 7, it can be seen that in the test cases (**a**), (**b**) and (**c**), the impinging limits are between −20 and 20 mm and in the test cases (**g**), (**h**) and (**i**) they are between −40 and 40 mm. This fact occurs because there are differences between the MVD of both (test cases (**a**), (**b**) and (**c**) have an MVD of 70 μm and (**g**), (**h**) and (**i**) cases have a MVD of 20 μm). Figure 8 shows that the ice forms farther from the leading edge in the case of large droplets. The FBGSs do not detect the larger impingement in SLD conditions because the turbulence generated by the ice accretion makes inaccurate the temperature predictions in the sensors located farther from the leading edge.

**Figure 9:**
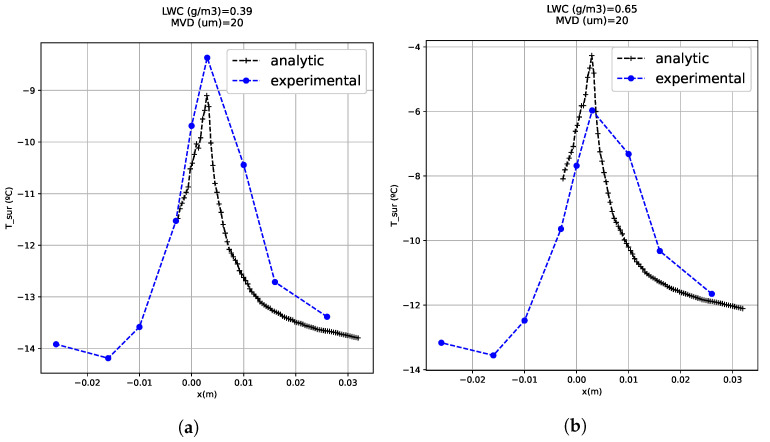

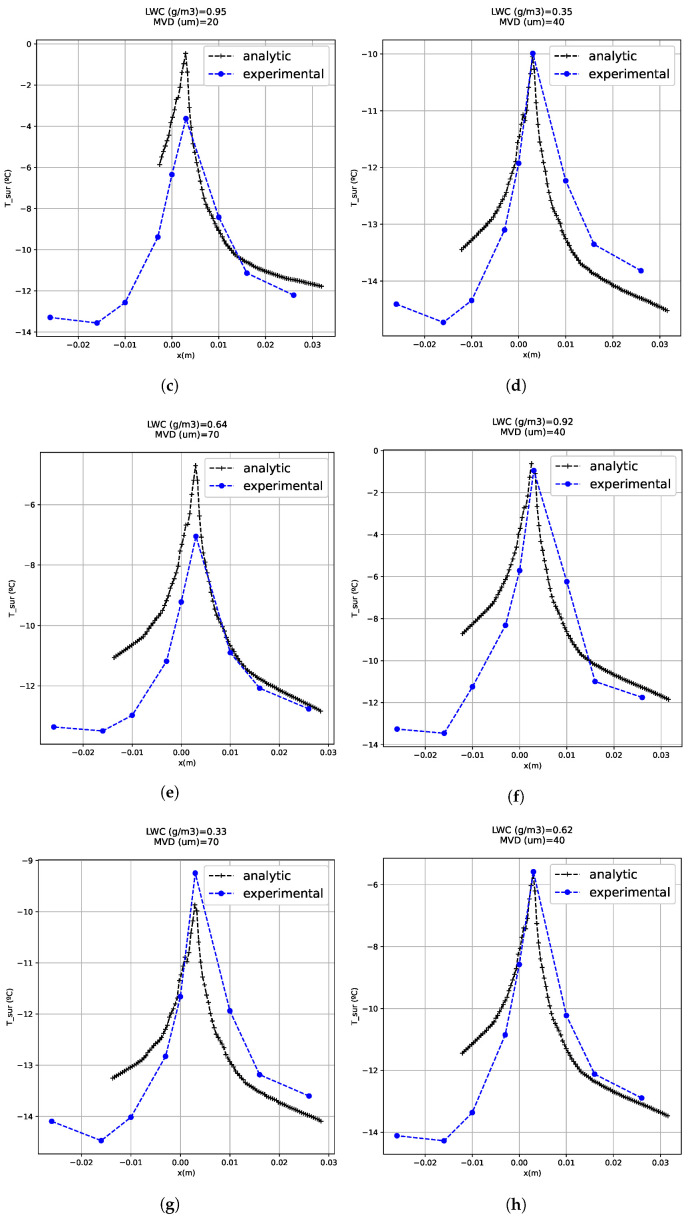

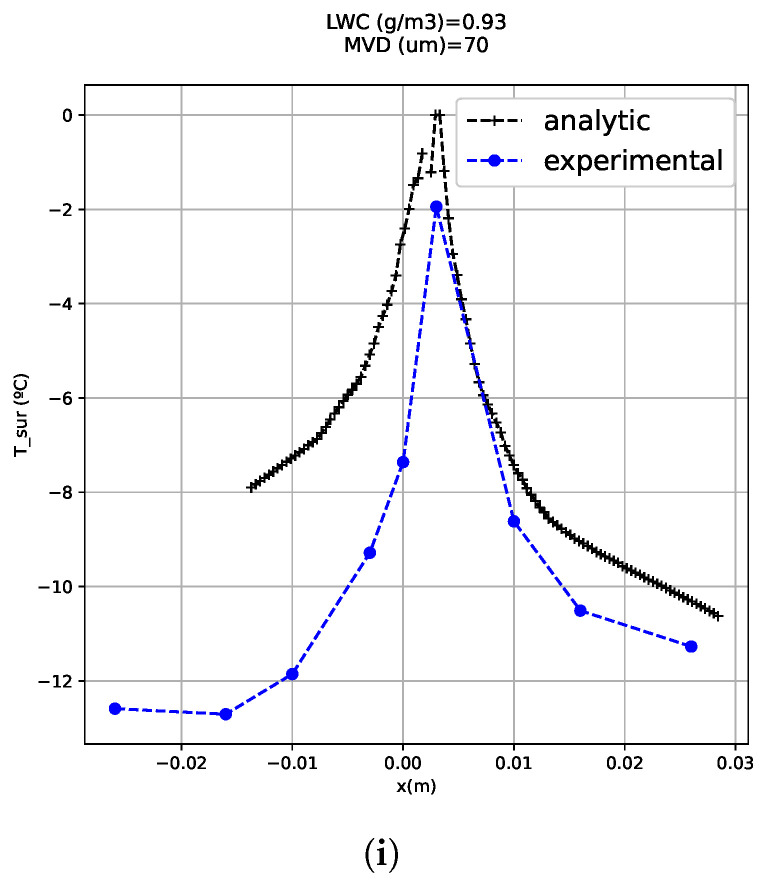
Rime test matrix. Angle of attack: −5${}^{\circ }$. Test cases: (**a**) 10 (**b**) 13 (**c**) 16 (**d**) 11 (**e**) 14 (**f**) 17 (**g**) 12 (**h**) 15 (**i**) 18.

There is a high accuracy of the model in rime ice with different angles of attack as well. For example, in Figure 9, several test results with minus five degrees of angle of attack are shown. The temperature distributions seem similar in the analytical and experimental results. This model can give an idea about where the point of maximum accretion is. In this case, it is in the airfoil upper surface, where the stagnation point on an airfoil with an angle of attack is located. There is a no symmetric ice accretion between the upper and lower surfaces due to the angle of attack. In the tests with higher liquid water contents, the equilibrium temperature is higher than with lower LWC (Figure 9c,f,i). There is an error which can be produced by a certain cloud non-uniformity. The non-uniformity of the icing cloud produces dispersion of ice accretion along the span of the airfoil, and high variability in the temperature profile.

**Figure 10:**
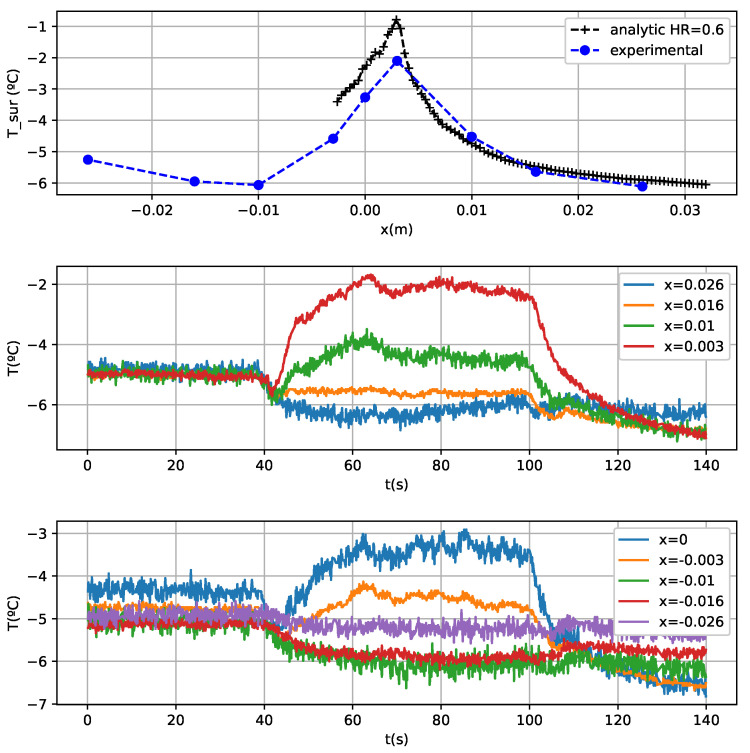
Test conditions: angle of attack: −5${}^{\circ }$. V = 70 m/s. LWC = 0.39 g/m${}^{3}$. MVD = 20 μm.

**Figure 11:**
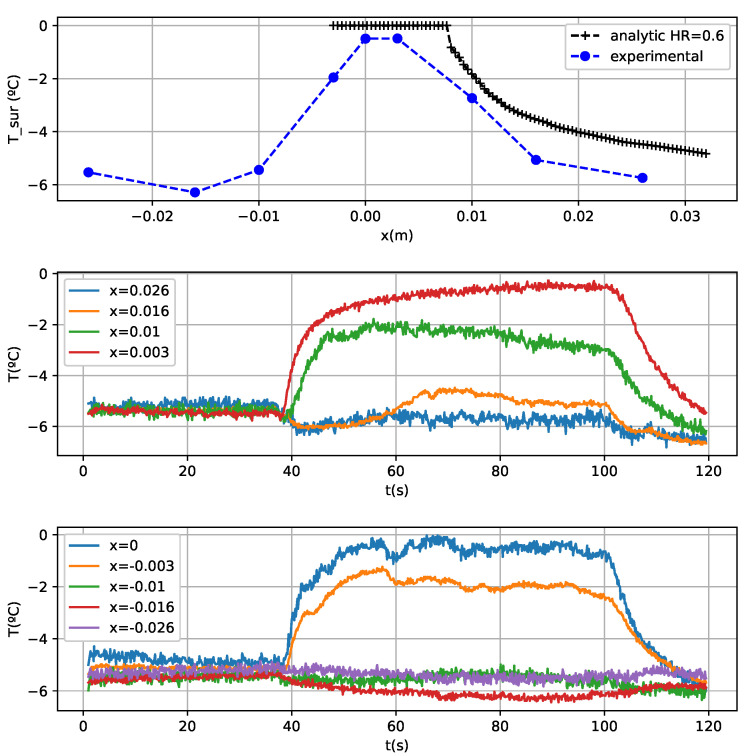
Test conditions: angle of attack: −5${}^{\circ }$. V = 70 m/s. LWC = 0.95 g/m${}^{3}$. MVD = 20 μm.

Glaze ice results are less accurate because in the applied model the evaporative term causes different solutions depending on the relative humidity of the air. Another problem of glaze ice tests is a temperature drop during the nebulization (Figure 10 and Figure 11). This could be an air cooling due to the supercooled water spray. Furthermore, there are equilibrium temperature errors probably because the leading edge sensors do not reach the equilibrium temperature (Figure 11) during the fogging cycle, so the experimental results are slightly lower than the analytical model. There is another source of error and there is a large required time for reaching the equilibrium temperature in some cases, for example, the sensor located 3 mm from the leading edge in Figure 11. Another difficulty in order to determine the equilibrium temperature can be seen in Figure 11 as well. For example, in the sensor located in the upper surface, in x = 0.01 m, there is a temperature rising to −2 ${}^{\circ }$C, and then a drop to −3 ${}^{\circ }$C. This could be produced by non-stationary effects

1. Initially, it was observed that there is more water fluency in the surface of the airfoil than in the end of the test. This could produce a more intense water freezing in the areas further away from the leading edge in the beginning of the test and less water freezing in the area close to it. This fact can be corroborated in Figure 11, in which initially the temperature of the grating located in x = 3 mm is similar to the temperature of the grating in x = 1 cm. This means that there is a similar latent energy realised in both gratings and a consequent similar ice accretion as well.
2. After a certain time, less water flowing was observed in the test, so there is more ice accretion in the area close to the leading edge. In Figure 11 can be observed a growth in the temperature difference between the sensors located in x = 3 mm and x = 1 cm. This corroborates the existence of water flow that was seen during the test. There is more ice formed in the leading edge, so there is less quantity of water running back and wetting the surface downstream and consequently less latent energy released in the back sensor.

The freezing fraction is the magnitude that measures the fraction of water that freezes as a function of the position. In the Figure 11 test it can be concluded that the freezing fraction is not stationary and changes during the test because the water accretion and fluency changes with time. In the case of rime tests, this effect cannot be seen because of the freezing fraction concerning the unity.

### 3.2. Convective Heat Coefficient in Rime Ice

One of the advantages of using FBGSs in rime ice is the easiness of the convective heat transfer coefficient calculation. Considering the sensors’ positions *i* as the nodes of the energy balance equation, it could be expressed as a function of the mass flux of impinging water, recovery temperature, specific heat of ice in the surface $c_{p,is}$, latent heat of fusion, specific heat of water $c_{pw}$, airspeed and surface profile detected by the sensors:

(24) $$h_{c}^{i}=\frac{{\dot{m}}_{im}^{i}}{T_{sur}^{i}-T_{rec}^{i}}\left(c_{p,is}^{i}\left(T_{sur}^{i}-T_{mp}\right)+\frac{V_{\infty }^{2}}{2}+L_{f}^{i}-c_{p,w}^{i}\left(T_{mp}-T_{\infty }\right)\right)$$

**Figure 12:**
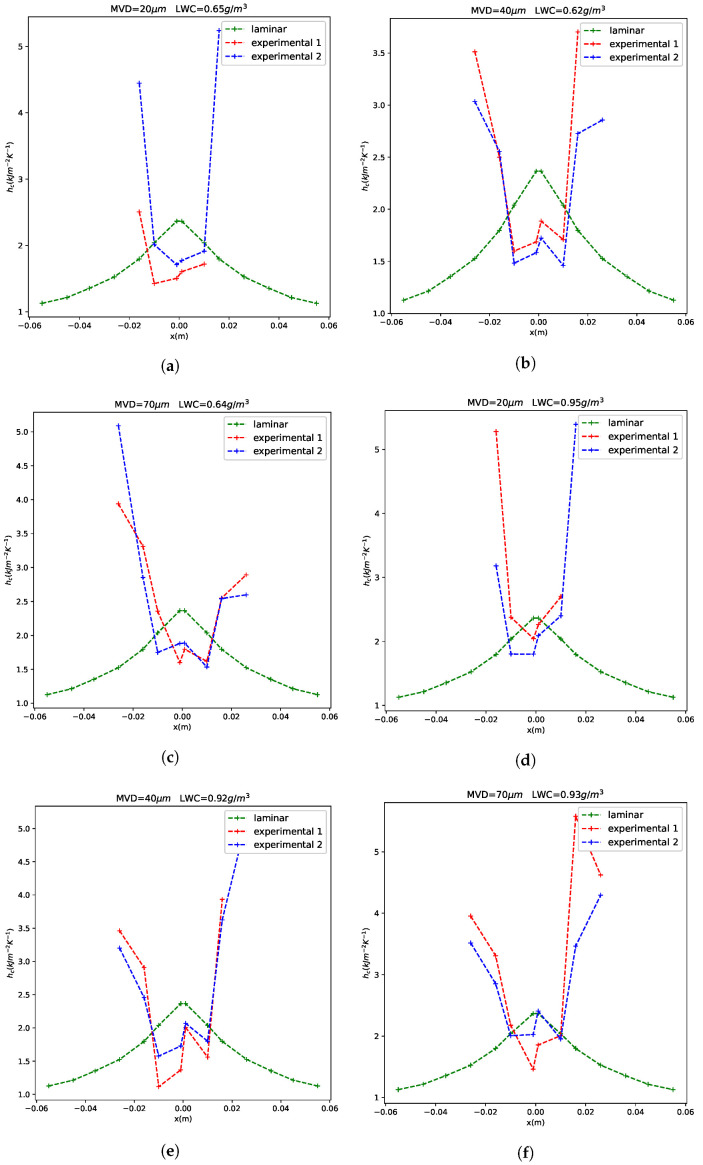
Convective heat transfer coefficient. Test cases: (**a**) 13 (**b**) 14 (**c**) 15 (**d**) 16 (**e**) 17 (**f**) 18.

The $T_{sur}^{i}$ are the FBGS values along the chord. The convective heat transfer coefficient values compared with the laminar solution in Section 2.2.2 can be seen in Figure 12. The value of the laminar heat transfer coefficient is accurate in the sensors located close to the leading edge because the flow is completely laminar. After a certain point, the flow begins to be turbulent and the convective heat transfer coefficient begins to depend on other parameters like the equivalent sand-grain roughness height or Reynolds number (K. Yamaguchi [12]). Figure 12 shows that the convective heat transfer coefficient could be approximated as constant in the laminar region. The variations in the laminar region are not as high as the differences of heat transfer coefficient between the laminar and the turbulent regions.

It is shown that FBGS can be a very useful tool in order to predict where the transition between laminar and turbulent flow is located. In this case, as can be seen in Figure 12, the laminar to turbulent transition occurs at approximately 1 cm from the leading edge. Other possible applications in the case of rime ice could be to determine an approximate quantification of the convective heat transfer coefficient magnitude in the turbulent side. A problem of quantifying the convective heat transfer coefficient using Equation (24) is the ignorance of the exact value of the impinging water mass flow. It is impossible to determine an exact value of impinging water because the icing cloud is not uniform in the test section, so the value is approximate.

## 4. Liquid Water Content Evaluation Algorithm

Once the test temperature has reached the equilibrium temperature (Figure 6), the temperature profile is represented along the chord. Knowing the true airspeed and the angle of attack, a CFD model could be calculated (see Section 2.2.1). The LWC prediction should be done using the sensors close to the stagnation point where the laminar flow is. The problem is that, in a real case, the droplet size of the population is unknown so the collection efficiency is impossible to calculate.

**Figure 13:**
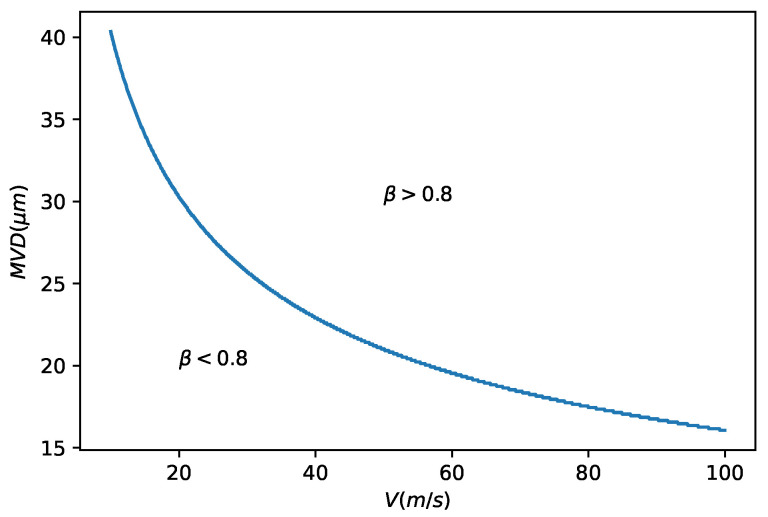
Collection efficiency in function of the airspeed and of the MVD.

For certain conditions, collection efficiency is higher than 0.8 so high LWC accuracy could be achieved. The conditions with high collection efficiencies were calculated using the method laid out in reference [27] and represented in Figure 13.

The LWC prediction procedure consists in calculating the impinging mass flow in the stagnation point sensor using the impinging water mass flow $m_{im}^{node}$. Using Equation (5) and simplifying it for the rime ice case, the impinging water mass flow is:

(25) $${\dot{m}}_{im}^{node}=\frac{h^{node}\left(T_{sur}^{node}-T_{rec}^{node}\right)}{c_{p,is}^{node}\left(T_{sur}^{node}-T_{mp}\right)+\frac{V_{\infty }^{2}}{2}+L_{f}^{node}-c_{pw}^{node}\left(T_{mp}-T_{\infty }\right)}$$

The collection efficiency of the problem is unknown so a $\beta_{0}=0.9$ is selected because it is between the minimum collection efficiency in the envelop $\beta_{0}=0.8$ and the maximum collection efficiency $\beta_{0}=1$. Once the impinging water has been calculated, the LWC is:

(26) $$LWC=\frac{{\dot{m}}_{im}}{V\beta_{0}}$$

The prediction results can be seen in Table 5. The errors are less than 20%. These errors could be interpreted as very high, but they are normal. According to the SAE 5905 [28], “the uniform icing cloud is defined as the area of the test section over which the LWC does not vary by more than ±20% from the test section centerline LWC value”. The sensor accuracy is inside the test error range, so the results are accurate.

In terms of modeling, glaze ice has the problem that the stagnation point temperature is 0 ${}^{\circ }$C, for whatever LWC. It has the additional disadvantage of relative humidity dependence (Messinger [6]), so using this method to predict glaze LWC is not accurate. While the exact LWC value cannot be predicted, an assessment of the minimum LWC could be done. If the equilibrium surface temperature of the airfoil reaches 0 ${}^{\circ }$C, the liquid water content is the one calculated from Equations (26) and (28) or higher. Another parameter that can be useful in order to know the LWC order of magnitude is the airfoil points where temperatures are 0 ${}^{\circ }$C. More airfoil surface with more than 0 ${}^{\circ }$C implies higher water flow on the surface and, therefore, more LWC.

### Ice Accretion Rate Algorithm

The accretion rate was calculated using the heat balance Equation (5). In rime ice, the impinging water mass flow calculation (Equation (28)) is necessary. Once the impinging water mass flow has been calculated, the ice accretion rate (dΔ/dτ) is:

(27) $$\frac{d\Delta }{d\tau }=\frac{{\dot{m}}_{im}}{\rho_{ice}}$$

Combining Equations (27) and (28), the ice accretion rate can be known:

(28) $$\frac{d\Delta }{d\tau }=\frac{h^{0}\left(T_{sur}^{0}-T_{rec}^{0}\right)}{\rho_{ice}\left(c_{p,is}^{0}\left(T_{sur}^{0}-T_{mp}\right)+\frac{V_{\infty }^{2}}{2}+L_{f}^{0}-c_{pw}^{0}\left(T_{mp}-T_{\infty }\right)\right)}$$

This variable does not depend on the liquid water content, the stagnation collection efficiency or the freezing fraction, so the value is expected to be more accurate. The total temperature of the test is the leading edge sensor temperature before nebulization. In Table 6 the ice accretion rates of different tests can be seen. The ice accretion rate is calculated dividing the ice thickness during the test with the test duration.

**Figure 14:**
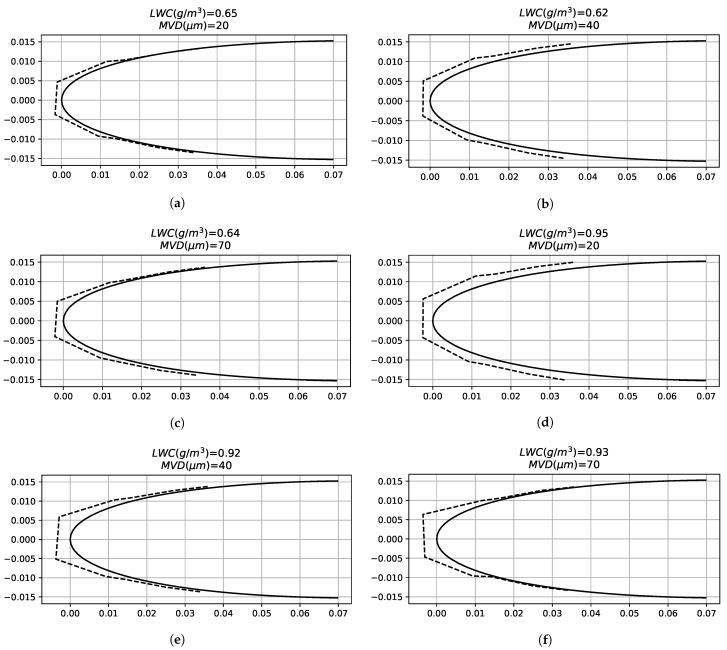
Ice accretion predictions. Test cases: (**a**) 13, (**b**) 14, (**c**) 15, (**d**) 16, (**e**) 17, (**f**) 18.

Using the ice accretion rate, it is possible to know approximately the ice geometry in the airfoil, applying Equation (28) in all sensors. The results of the ice accretion prediction can be seen in Figure 14. The results seem accurate for rime ice accretion.

## 5. Conclusions

An optic fiber with eight FBGSs was integrated in each of the upper and lower surfaces of a cut NACA 0012 aerodynamic profile in order to measure the temperature in sixteen points along its chord. An icing test campaign was performed in the INTA IWT, some of them in hazardous SLD conditions. Analytical and experimental results, using a Messinger heat and mass balance model and the FBGS measurements show a good correlation of the theoretical and empiric data. The icing prediction temperatures obtained with the integrated FBGSs along the chord are quite accurate near the leading edge, demonstrating that these optical sensors enable fast and precise detection of the beginning and the end of the icing hazard, the evaluation of the liquid water content and the ice accretion rate. Downstream from the leading edge an increment of errors occur that are caused by the aerodynamic turbulence. The LWC predictions obtained in the test campaign show a maximum error of 20% for Table 3 icing cloud conditions. The ice accretion rate presents acceptable results as well.The uncertainties that are obtained may be caused by not knowing cloud non-uniformity, collection efficiency or the heat transfer coefficient.

Finally, it is important to note that the integrated fiber optic sensors can be used not only for the ice detection and icing condition evaluation in aircrafts, which is what they were initially developed for, but can help also to know and properly interpret some physical events during IWT tests. Knowing the surface temperature during an icing test can give much information about the heat transfer events and, therefore, the ice accretion in the airfoil surface in the first moments of the flogging cycles. An integration of the tiny fiber optic sensors in these IWT test models can be carried out normally without a significant influence in its aerodynamic performance.

## Figures and Tables

**Table 1:** Calibration accuracy.

Temperature (${}^{\circ }$C)	20	10	0	−10	−20	−30
${\mathrm{acc}}_{T}$ **(pm)**	1.1	1.1	1.0	1.2	1.0	1.1
acc **(** ${}^{\circ }$ **C)**	0.1	0.2	0.2	0.2	0.2	0.2

**Table 2:** CFD model parameters.

Airspeed (m/s)	Inlet	Free stream velocity
	Outlet	Free stream velocity
	Airfoil walls	No slip
Kinematic Pressure (Pa)	Inlet	free stream Pressure
	Outlet	free stream Pressure
	Airfoil walls	Zero Gradient
Turbulent kinematic viscosity (m${}^{2}$s${}^{-1}$)	Inlet	≈$\nu_{fluid}$
	Outlet	≈$\nu_{fluid}$
	Airfoil walls	0
Spalart-Allmaras model modified viscosity (m${}^{2}$s${}^{-1}$)	Inlet	≈$4\nu_{fluid}$
	Outlet	≈$4\nu_{fluid}$
	Airfoil walls	0

**Table 3:** Test Matrix. (**a**) $T_{t}\approx 5$ ${}^{\circ }$C. (**b**) $T_{t}\approx 13.5$ ${}^{\circ }$C.

Test	MVD	LWC	Test	MVD	LWC
Number	(μm)	(g/m${}^{3}$)	Number	(μm)	(g/m${}^{3}$)
1	20	0.39	10	20	0.39
2	40	0.35	11	40	0.35
3	70	0.33	12	70	0.33
4	20	0.65	13	20	0.65
5	40	0.63	14	40	0.63
6	70	0.64	15	70	0.64
7	20	0.95	16	20	0.95
8	40	0.92	17	40	0.92
9	70	0.93	18	70	0.93
(**a**)			(**b**)		

**Table 4:** Chordwise distance of each sensor from the leading edge.

Sensor	x (mm)	Sensor	x (mm)
A8	0	A16	−0
A7	3	A15	−3
A6	10	A14	−10
A5	18	A13	−18
A4	27	A12	−27
A3	35	A11	−35
A2	46	A10	−46
A1	56	A9	−56
(**a**) Upper surface sensors		(**b**) Lower surface sensors	

**Table 5:** Icing blade and predicted LWC comparison.

Predicted LWC (g/m${}^{3}$)	Icing Blade LWC (g/m${}^{3}$)	Error (%)	MVD (μm)
0.46	0.39	18.55	20
0.76	0.65	16.27	20
0.68	0.65	4.89	20
0.79	0.95	16.57	20
0.87	0.95	8.52	20
0.38	0.35	8.73	40
0.63	0.62	2.36	40
0.7	0.62	12.6	40
0.95	0.92	3.04	40
0.89	0.92	2.8	40
0.39	0.33	19.05	70
0.73	0.64	13.88	70
0.69	0.64	7.32	70
1.09	0.93	17.08	70
0.82	0.93	11.98	70

**Table 6:** Ice Accretion Rate prediction.

MVD	LWC	Analytical Accretion	Measured Accretion	Error
(μm)	(g/m${}^{3})$	Rate (mm/min)	Rate (mm/min)	(%)
20	0.39	1.07	1.05	2.0
20	0.65	1.79	1.57	14.3
20	0.95	2.51	3.05	17.8
40	0.35	0.94	1.05	11.0
40	0.62	1.7	1.77	3.8
40	0.92	3.64	3.07	18.9
70	0.33	1.04	1.21	13.7
70	0.64	2.31	1.97	17.2

## Data Availability

Not applicable.

## Abbreviations

The following abbreviations are used in this manuscript:

CFD	Computational Fluid Dynamics
FBGS	Fiber Bragg Grating Sensor
IAR	Ice Accretion Rate
IWT	Icing Wind Tunnel
LWC	Liquid Water Content (g m${}^{-3}$)
MVD	Median Volume Diameter (μm)
SLD	Supercooled Large Droplets

## Nomenclature

$c_{p,a}$	Air specific heat (J m${}^{-2}$ kg${}^{-1}$)
$c_{p,i}$	Ice specific heat (J m${}^{-2}$ kg${}^{-1}$)
$c_{p,w}$	water specific heat (J m${}^{-2}$ kg${}^{-1}$)
$D_{a,v}$	Mass diffusivity (m${}^{2}$/s)
*f*	freezing fraction
$h_{con}$	Convective heat transfer coefficient (W m${}^{-2}$ K${}^{-1}$)
$h_{g}$	Mass Transfer coefficient (m/s)
$k_{a}$	Air thermal conductivity (W K${}^{-1}$ m${}^{-1}$)
$L_{lat}$	water latent energy of fusion (J/kg)
$L_{v}$	Latent heat of vaporization (J/kg)
Le	Lewis number
LWC	Liquid Water Content (g/m${}^{3}$)
${\dot{m}}_{ice}$	mass flux of ice formed in the surface (kg m${}^{-2}$ s${}^{-1}$)
${\dot{m}}_{im}$	mass flux of water that impinges the surface (kg m${}^{-2}$ s${}^{-1}$)
${\dot{m}}_{rb}$	mass flux that enters in the control volume (kg m${}^{-2}$ s${}^{-1}$)
${\dot{m}}_{out}$	the mass flux that leaves that control volume (kg m${}^{-2}$ s${}^{-1}$)
${\dot{m}}_{evap}$	the mass flux of water that is evaporated (kg m${}^{-2}$ s${}^{-1}$)
$P_{vs}$	Saturated Vapour pressure (Pa)
$P_{st}$	Static Pressure (Pa)
$q_{lat}$	impinging water gained Fusion heat flux (W m${}^{-2}$)
$q_{evap}$	Water evaporation loss heat flux (W m${}^{-2}$)
$q_{sens}$	Sensible heat flux (W m${}^{-2}$)
$q_{k}$	Kinetic droplet gained heat flux (W m${}^{-2}$)
$q_{nc}$	Net convective heat flux (W m${}^{-2}$)
$q_{c}$	Convective droplet heat flux (W m${}^{-2}$)
$q_{fric}$	frictional gained heat flux (W m${}^{-2}$)
*s*	Surface distance (m)
$T_{st}$	Static temperature (K)
$T_{mp}$	Melting point temperature (K)
$T_{rec}$	Recovery temperature (K)
$T_{sur}$	Surface temperature (K)
$V_{e}$	Airspeed in the exterior of the boundary layer (m/s)
$V_{\infty }$	Airspeed (m/s)
*x*	Horizontal distance (m)

## Greek Letters

α	thermal expansion (1/K)
β	Collection efficiency
$\delta_{T}$	Thermal boundary layer (m)
Δ	Ice thickness (m)
$\lambda_{B}$	Reflected wavelength (nm)
$\mu_{a}$	dinamic viscosity (N s m${}^{-2}$)
$\nu_{a}$	kinematic viscosity (m${}^{2}$ s${}^{-1}$)
$\rho_{a}$	Air density (kg/m${}^{3}$)
$\rho_{vs}$	Water Vapor density at the surface (kg/m${}^{3}$)
$\rho_{ve}$	Water Vapor density in the air (kg/m${}^{3}$)
τ	time (s)
ϕ	Relative humidity
